# Editorial

**Published:** 1872-05

**Authors:** 


Our April Number was delayed by causes which we hope will not happen again. Owing to this delay, the last form of that number was worked off so hurriedly that the proof sheets of our editorials did not receive that careful reading to free them from the many typographical errors with which they were disfigured. An unusual press of business on the part ot the editors, the sickness of the foreman of the office at the time, and the hurried publication above mentioned, must be our apology. With the generous, sensible reader this will, doubtless, be sufficient.

Our Senior is absent, being in attendance upon the meeting of the American Medical Association. He will doubtless give his "*experiences*" in our next.

Cerebro-Spinal Meningitis is prevailing in almost every portion of the Union, but seems nowhere to have assumed an epidemic character. New suggestions as to treatment are being made in almost every section ; and we trust many points of value may be evolved for the lessening of the great fatality of this fearful malady. Our friend and fellow-townsman, Dr. J. J. Knott, has had good success in its management by the use of calomel, bromide of potassium and quinine in doses many would regard as "heroic." We trust that he will give his views to the profession, as he and others have, in carrying out his method, received excellent results. Dr. E. N. Calhoun, of this city, advocates tobacco enemata, and is convinced, from several cases he has treated in this manner, that it will prove the best treatment as a relaxant. It has been used successfully, it is affirmed, in other portions of the country. W. T. G.

Hooping-Cough.—Our city has been visited by an epidemic of this disease. We mention the fact with the view of calling attention to two methods of treatment, which, in our hands, have been *entirely* satisfactory by being *entirely* successful. In the first, or catarrhal stage, we have employed an old formula—which, from this fact, has been called a "domestic" remedy in the U. S. Dispensatory—to-wit: the carbonate of potass. and cochineal. We were induced to use it at the suggestion and recommendation of Dr. E. N. Calhoun, of this city, and have found it to come fully up to his statements concerning it. In every case in which we have used these drugs, in the incipiency of the disease, it has either aborted, or so modified it, as to entirely prevent the "hoop" so characteristic of the affection. The catarrhal stage preceeds that of the "hooping" eight or ten days, and if the remedy be used constantly and persistently during this stage, our experience justifies us in asserting that the disease will be so broken up or modified as never to reach the latter—that of "hooping." Given later, it has some control over the "hooping stage," but not more, and we think less, than other remedies. It is only in the first stage of the disease that its truly marvellous virtues are exhibited. We used the following formula:

| | |
|---|---|
| R.—Carbonate of Potass., . . . . . | grs.40. |
| Cochineal, finely powdered, . . . . : | grs.20. |
| Water, . . . . . . . | oz.8.—M. |

S.—A teaspoonful to an infant less than one year old; two teaspoonfuls to children of two or three years, and three or four teaspoonfuls to children of four to six years of age, four times a day. Each dose should be made thoroughly sweet with the best loaf sugar every time it is given.

The second stage has been controlled more successfully in our hands by the employment of *benzine* than any other remedy. Ten or more drops are to be given internally through the day, and the atmosphere of the sleeping apartment rendered "gassy" by its vapor, the benzine being sprinkled on the pillow and bed-covering of the patient. It should be used cautiously at night, as it is extremely inflammable—igniting readily some distance from a flame. W. T. G.

